# Comparative Amplicon and Shotgun Metagenome Profiling of Soil Microbial Communities in Kauri Forests Affected by *Phytophthora agathidicida*

**DOI:** 10.1111/1758-2229.70324

**Published:** 2026-04-01

**Authors:** Zoe King, Hannah L. Buckley, Gavin Lear, Brent Seale, Kevin C. Lee, Luitgard Schwendenmann, Donnabella C. Lacap‐Bugler

**Affiliations:** ^1^ School of Science Auckland University of Technology Auckland New Zealand; ^2^ School of Biological Sciences The University of Auckland Auckland New Zealand; ^3^ School of Environment The University of Auckland Auckland New Zealand

**Keywords:** amplicon sequencing, kauri dieback, LAMP detection, pathogen‐microbe interactions, *Phytophthora agathidicida*, shotgun metagenomics, soil microbiome

## Abstract

Soil‐borne pathogens can influence microbial communities and ecosystem function, making it important to understand their broader ecological impacts. We investigated interactions between *Phytophthora agathidicida* (the causal agent of kauri tree dieback) and soil microbial communities, while also comparing detection and community‐profiling methods. Soils from 60 kauri trees across three sites in the Waitākere Ranges, New Zealand, were analysed using loop‐mediated isothermal amplification (LAMP) for pathogen detection, and 16S rRNA gene/ITS gene amplicon sequencing alongside shotgun metagenomics for community characterisation. LAMP detected *P. agathidicida* in 39/60 samples, while shotgun sequencing detected *Phytophthora*‐associated DNA at low abundance across all samples. Microbial community structure and functional potential showed weak association with pathogen presence, though differential abundance testing identified several genera enriched in pathogen‐detected soils, including taxa previously linked to disease suppression. Amplicon and shotgun profiles indicated broadly comparable patterns at higher taxonomic and functional levels, while differences between approaches emerged primarily at finer taxonomic resolution. Importantly, functional predictions from PICRUSt2 closely matched shotgun‐derived profiles at broader scales, indicating its suitability as a cost‐effective tool for broad‐scale monitoring. These findings suggest limited direct pathogen effects on microbial communities and highlight how integrating molecular approaches provides complementary insights into soil microbiome‐pathogen interactions.

## Graphical abstract

Soils beneath symptomatic and asymptomatic kauri trees were analysed using LAMP, amplicon sequencing, shotgun metagenomics, and PICRUSt2. LAMP detected *Phytophthora agathidicida* in 39/60 samples, while shotgun metagenomics detected it at low abundance across all samples. Microbial communities showed weak association with pathogen presence but subtle enrichment of some disease‐suppressive taxa.

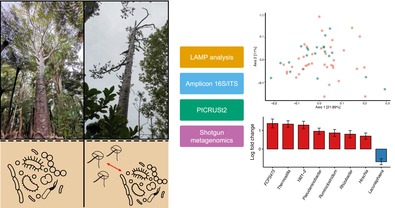

## 1 Introduction

*Phytophthora agathidicida*, the causal pathogen of kauri dieback, is a soil‐borne oomycete that poses a significant threat to *Agathis australis* (kauri), a culturally and ecologically significant foundation tree species in New Zealand (Beever et al. 2009; Ecroyd 1982; Weir et al. 2015). The lifecycle of *P. agathidicida* involves phases of both dormancy and infection, where it may survive in the soil for extended periods through the production of oospores (Bradshaw et al. 2020; Weir et al. 2015). Upon sensing root‐derived chemical signals from kauri, the pathogen breaks its dormancy stage, producing motile zoospores that travel towards the roots and initiate infection (Bradshaw et al. 2020). Disease progression is characterised by root rot, a thinning canopy, and basal bleeding/gummosis on the trunk or lateral roots, which eventually leads to tree death (Beever and Bellgard 2010; Bellgard et al. 2016; Waipara et al. 2013). Understanding how the presence of *P. agathidicida* influences the surrounding soil microbial community is important for assessing the wider ecosystem impacts of kauri dieback and loss of this foundation tree species. The partially free‐living but primarily pathogenic lifecycle of *P. agathidicida* suggests that the pathogen may affect the soil microbiome through direct interactions or indirectly via host‐mediated effects (Feng et al. 2024; Reverchon et al. 2023; Tong et al. 2024).

Various methods are used to detect and monitor *P. agathidicida*, describe and quantify disease expression in trees, characterise the soil microbiome, and assess the functional potential of the microbial community. These include visual assessment of symptoms, soil baiting and culturing followed by morphological identification, and molecular‐based techniques such as loop‐mediated isothermal amplification (LAMP), amplicon sequencing, and shotgun metagenomic sequencing, respectively (Beever and Bellgard 2010; Byers, Condron, O'Callaghan, et al. 2020; Dick and Bellgard 2010; Hill et al. 2017; Sivaprakasam et al. 2024; Winkworth et al. 2020). Determining whether the outputs of these different approaches are related will provide important context for interpreting results and support the methods and approaches used in subsequent studies.

Although *P. agathidicida* detection is an essential component of disease management, it remains challenging, despite recent advancements. Visual assessment of kauri dieback symptoms is widely used in field surveillance, but its sensitivity is limited (Froud et al. 2022). Aboveground symptoms can develop long after the pathogen first infects the roots, and even when visible, they may reflect other causes such as infection by other *Phytophthora* species rather than *P. agathidicida* specifically or drought (Beever and Bellgard 2010; Froud et al. 2022; Hunter et al. 2024; Waipara et al. 2013).

A symptomatic tree is defined as having at least one of the following symptoms: some branch dieback, gummosis on the trunk base or lateral roots, colour change of leaves to yellow or copper‐brown, or tree death (Bellgard et al. 2013; Froud et al. 2022). Although more specific than visual assessment, the enrichment, culturing, and morphological identification of *P. agathidicida* is laborious, expensive, and prone to generating false negative results due to uneven pathogen distribution and difficulties in breaking oospore dormancy (Bellgard et al. 2013). The inclusion of molecular‐based methods with *P. agathidicida* enrichment, using tools such as LAMP assays (Winkworth et al. 2020), has improved sensitivity for pathogen detection, and metabarcoding techniques offer further resolution (Hunter et al. 2024); however, each method has its limitations in terms of cost, scalability, and context‐dependence.

Limited studies have investigated how *P. agathidicida* impacts soil microbial communities surrounding kauri. Existing research has primarily focused on describing microbial community differences between symptomatic and asymptomatic trees, using amplicon‐based profiling for bacterial (16S rRNA gene) and fungal (internal transcribed spacer (ITS) region) communities (Byers, Condron, Donavan, et al. 2020; Byers et al. 2021), or exploring microbial functional potential through microarray methods such as GeoChip (Byers, Condron, O'Callaghan, et al. 2020; Lawrence et al. 2023). While these studies have provided initial insights into the microbial communities, none have explicitly examined how the soil microbial community structure or function may be impacted by the confirmed presence or absence of *P. agathidicida* in soil. Additionally, no studies have applied shotgun metagenomic sequencing to kauri forest soils impacted by kauri dieback, which could increase taxonomic and functional resolution compared to previous methods.

Traditional culture‐based methods have been used extensively to study microbial communities, but they are only able to capture a small fraction of the true diversity present in most natural environments as the majority of microbes are not readily cultured (Daniel 2005; Nwachukwu and Babalola 2022). In contrast, culture‐independent sequencing approaches, particularly shotgun metagenomics, extract and sequence total environmental DNA, allowing for comprehensive taxonomic profiling and functional inference without the biases introduced by cultivation or targeted amplification (Edwin et al. 2025; Nwachukwu and Babalola 2022). Recent studies have used these approaches to recover metagenome‐assembled genomes (MAGs), resolve microbial community structure at fine taxonomic scales, and explore metabolic potential within forest soil environments (Cha et al. 2025; Midot et al. 2025; Qiao et al. 2025).

Beyond exploring microbial diversity and discovering novel taxa and enzymes, shotgun metagenomics has become a common tool in environmental and plant‐associated microbiome research. For example, metagenomic sequencing has been used to elucidate plant‐microbiome interactions, revealing how microbial communities influence plant health, nutrient cycling, and resistance to pathogens (Huang et al. 2024; Kazarina et al. 2025; Wani et al. 2025). These applications highlight the value of metagenomic approaches for capturing complex community structure and functional potential, offering a richer and more complete view of soil microbiomes than culture‐based or marker gene methods. In the context of plant health and disease ecology, comprehensive genetic studies are particularly valuable for identifying co‐occurring taxa with pathogens and inferring possible functional interactions that may influence disease outcomes. This is important for soilborne pathogens like *P. agathidicida*, where a detailed view of the surrounding microbial community may inform links between microbial context and pathogen presence or activity.

In the presence of a plant pathogen, soil microbial communities may be affected by both direct and indirect interactions. Direct interactions include microbial competition with *P. agathidicida* for nutrients or niche space and the production of antimicrobial compounds that suppress pathogen activity (Chen et al. 2018; Gu et al. 2020). Indirect interactions may arise through changes in plant physiology in response to infection, such as increased litterfall or gummosis, which alter nutrient input into the surrounding soil environment (Avila et al. 2016; Delgado‐Baquerizo et al. 2016), or shifts in root exudation profiles to recruit specific microbial taxa, including those with biocontrol potential (Köhl et al. 2019; Wang et al. 2021). These complex and bidirectional interactions involve antagonistic and compensatory processes that directly affect plant health and disease expression. As infection progresses, these shifts may create new ecological niches that enable opportunistic organisms, such as saprotrophs or secondary pathogens, to proliferate and further disrupt the native microbial community (Byers, Condron, O'Callaghan, et al. 2020; Gómez‐Aparicio et al. 2022; Jung et al. 2018).

This study compares the various methods used to detect and measure the relationship between the presence of *P. agathidicida* and the structure and function of kauri forest soil microbial communities. We ask: (1) How do the detection rates of *P. agathidicida* using LAMP, amplicon sequencing, and shotgun metagenomic sequencing compare? (2) How is the soil microbial community structure (diversity and composition) related to the confirmed presence of *P. agathidicida*, as assessed by amplicon and shotgun sequencing? (3) How is the potential soil microbial community function related to the confirmed presence of *P. agathidicida*? and (4) How do the functional and taxonomic profiles derived from shotgun metagenomic profiling align with those inferred from 16S rRNA gene amplicon sequencing? Investigating these questions will improve our understanding of how microbial community structure and function are related to the confirmed presence of a plant pathogen. Further, this study provides insights into the reliability, complementarity, and limitations of two high‐throughput sequencing techniques in detecting microbial community signatures associated with plant pathogen presence.

## 2 Experimental Procedures

### 2.1 Field Sampling

Samples were collected from the Waitākere Ranges (Te Wao Nui ā Tiriwa), a regional park containing one of the largest remaining kauri forests in Auckland, New Zealand. Three sites within the Waitākere Ranges were selected for sampling: the Cascades, Piha, and Huia (Figure S1) (*n* = 3 sites). Within each site were two permanent vegetation plots established between 2012 and 2021 (*n* = 6 plots). Each plot was 40 × 50 m, and all kauri trees with a minimum diameter at breast height of 2 cm were identified and tagged at the plot's establishment. Ten kauri trees were selected for soil sampling in each plot, consisting of four trees at the corner of each plot and six other kauri trees randomly selected from within the plot (*n* = 60). Following an established protocol for soil sampling (Hill et al. 2017), woody and leaf litter were removed to expose soil before a hand trowel was used to collect soil from four cardinal points around each tree, 1 m from the trunk to a depth of 10 cm. The cardinal point soil samples were pooled into one bag before being transferred to a −20°C freezer and stored until processing. Soil sample collection was conducted by BioSense Limited (Auckland, New Zealand) in February and March of 2022. Canopy scores were recorded for all sampled trees using an established five‐point scoring system (1 = good condition, 2 = some foliar thinning, 3 = some branch dieback, 4 = severe shoot dieback, and 5 = dead) following Horner et al. (2019), with additional half‐point increments included to provide greater differentiation, particularly in the later stages of infection (Froud et al. 2022).

### 2.2 Soil DNA Extraction, Amplicon and Shotgun Metagenome Sequencing

DNA was extracted from the 60 soil samples using a DNeasy PowerSoil Pro Kit (Qiagen, Germany), following the manufacturer's instructions, using 0.25 g of soil for each sample. Negative controls using nuclease‐free water were included in each batch of extractions (*n* = 14), and DNA concentration was determined fluorometrically using the Qubit double‐stranded DNA BR assay kit (Thermo Fisher Scientific, Massachusetts, USA).

For amplicon sequencing, samples with a DNA concentration over 200 ng/μL were diluted with an equal volume of nuclease‐free water to reduce the amount of starting material in the PCR reaction.

Extracted DNA was amplified using the prokaryotic 341F (TCGTCGGCAGCGTCAGATGTGTATAAGAGACAGCCTACGGGNGGCWGCAG) and 805R (GTCTCGTGGGCTCGGAGATGTGTATAAGAGACAGGACTACHVGGGTATCTAATCC) primers to target the V3‐V4 region of the 16S rRNA gene (Stoeck et al. 2010). The ITS1 region of the fungal ITS region was amplified using the ITS1‐F (TCGTCGGCAGCGTCAGATGTGTATAAGAGACAGCTTGGTCATTTAGAGGAAGTAA) (Gardes and Bruns 1993) and ITS2 (GTCTCGTGGGCTCGGAGATGTGTATAAGAGACAGGCTGCGTTCTTCATCGATGC) (White et al. 1990) primers. Target‐specific sequences are underlined; the remaining primer sequence consists of Illumina adaptor sequences necessary for downstream analysis.

Each 25 μL reaction contained 6.25 μL of KAPA HiFi Hotstart ReadyMix (Kapa Biosystems, Wilmington, MA, USA) and 2 μL of template. For 16S rRNA gene amplification, 0.75 μL of each primer (10 μM) was included per reaction, and for the ITS region amplification, 1 μL of each primer (10 μM) was included. Thermocycler conditions were the same for both primer sets. They were as follows: initial denaturation at 95°C for 3 min, followed by 30 cycles of 98°C for 20 s (denaturation), 63°C for 15 s (annealing), and 72°C for 15 s (extension), with a final extension at 72°C for 1 min.

PCR products were purified using AMPure XP beads (Beckman Coulter, Auckland, NZ) following the manufacturer's instructions. Purified products were quantified using a Qubit double‐stranded DNA HS assay kit (Thermo Fisher Scientific, MA, USA) and normalised to 1 ng/μL. Samples were indexed (Nextera XT DNA Library Prep kit; Illumina, CA, USA), pooled, and purified using AMPure XP beads (Beckman Coulter, Auckland, NZ) following the manufacturer's instructions. Libraries were pooled at 1 nM, validated using an Agilent 2100 expert High Sensitivity DNA Bioanalyzer assay (Agilent Technologies, CA, USA), and sequenced using an Illumina MiSeq Reagent Kit v3 (600‐cycle) to produce 2 × 300 bp reads.

For shotgun metagenome sequencing, extracted DNA was normalised to 10 ng/μL before sending to Livestock Improvement Corporation (LIC; Hamilton, New Zealand) for library preparation and sequencing on an Illumina NovaSeq 6000 system using an S4 flow cell, producing sequence lengths of 2 × 150 bp. Two samples failed sequencing on the S4 flow cell and were re‐sequenced using an SP 300 flow cell. The SP 300 flow cell produced over double the number of sequences per sample compared to the S4 flow cell. To maintain a similar number of sequences per sample, 60 million sequence reads from each set of forward and reverse reads were randomly subset from the raw re‐sequenced samples using seqtk (https://github.com/lh3/seqtk).

### 2.3 Phytophthora Detection

The detection of *P. agathidicida* in each of the 60 soil samples was assessed by an initial baiting enrichment assay following the protocol described by Struijk et al. (2024) before total DNA was extracted from the cedar needle baits and analyzed using the LAMP assay described by Winkworth et al. (2020). This assay was performed by BioSense Limited (Auckland, New Zealand).

High‐throughput sequencing data from amplicon and shotgun metagenomic sequencing were analysed for *P. agathidicida* DNA. For the amplicon dataset, amplicon sequence variants (ASVs) were inferred from ITS1 sequences using DADA2 v1.24.0 (Callahan et al. 2016). To explore whether *P. agathidicida* could be detected within this fungal community dataset, fungal ASVs were compared against a custom reference database using BLASTn via BLAST v2.16 (Camacho et al. 2009). The reference database was constructed from ITS sequences from *P. agathidicida* isolates (GenBank accessions: JX122749.1, (Than et al. 2013), KP295308.1, KP295314.1, KP295312.1, and KP295311.1 (Weir et al. 2015)) using the *makeblastdb* (−dbtype nucl) function with default parameters. BLASTn searches were run with the following parameters: evalue 1e‐10‐word_size 24‐perc_identity 97‐qcov_hsp_perc 90‐dust no. To determine whether any ASVs belonged to the *Phytophthora* genus or Oomycota phylum, broader taxonomic classification of ASVs was performed using the UNITE “all eukaryotes” database v8.3 (Abarenkov et al. 2021), which includes 140 reference sequences from the genus *Phytophthora* but no specific reference sequence for *P. agathidicida*. To assess whether the commonly used fungal ITS1 primers (ITS1‐F and ITS2) are capable of amplifying *P. agathidicida*, primer binding was assessed against the *P. agathidicida* reference genome (strain 3770, chromosome 10; GenBank accession: CP106980.1; positions: 57,500‐58,500). The ITS1‐F and ITS2 primers are designed to anneal to conserved regions of the 18S and 5.8S rRNA genes, respectively, thereby amplifying the ITS1 region between them (Gardes and Bruns 1993; White et al. 1990). Primer‐template alignment was performed using BLASTn‐short (BLAST v2.16) which is designed for short sequence matching (Madden and Camacho 2008). Searches were conducted with the parameters −task blastn‐short, −strand both, −word_size 7, and −evalue 1000.

A custom Kraken2 database was constructed for shotgun metagenomic analysis using all NCBI Taxonomy entries associated with “Oomycota”, Taxonomy ID: 4762. Shotgun metagenomic reads were quality filtered, and human‐associated reads were removed before taxonomic classification using Kraken v2.1.2 (Wood et al. 2019) against the custom oomycota database (described in more detail below). Read counts were refined using Bracken v2.7 (Lu et al. 2017) to estimate the abundance of *P. agathidicida*‐associated DNA across samples. In this study, “*P. agathidicida*‐associated DNA” refers to shotgun metagenomic reads classified as *P. agathidicida* by Kraken2 against the custom reference database. This terminology acknowledges that taxonomic assignments from short‐read classification may include sequences from closely related taxa or extracellular DNA and therefore does not confirm the presence of viable *P. agathidicida* cells.

### 2.4 Bioinformatics

#### 2.4.1 Amplicon Data

Raw sequence reads underwent adapter removal using Cutadapt v4.4 (Martin 2011), with a minimum length threshold of 200 bp and 20 bp for the 16S and ITS reads, respectively; untrimmed reads were discarded. Amplicon sequence variants were generated using DADA2 implemented in R v4.4.0 (R Core Team 2021). Due to the poor quality of the reverse reads, only the forward reads were analysed (Pauvert et al. 2019; Ramakodi 2021). ASV taxonomy was inferred using the naïve Bayesian classifier method (Wang et al. 2007) against the SILVA database NR99 v138.1 (Quast et al. 2012) for 16S ASVs and the UNITE “all eukaryotes” database v8.3 (Abarenkov et al. 2021) for ITS ASVs. Non‐bacterial and non‐fungal ASVs were removed from the 16S and ITS datasets, respectively. Samples with < 1000 reads were removed from the datasets, resulting in the loss of two samples from the 16S dataset and three samples from the ITS dataset (Table S1). Reads were decontaminated using the *isContaminant* function in the R ‘decontam’ package v1.24.0 using the prevalence method and a threshold value of 0.5 (Davis et al. 2018). Briefly, the prevalence method identifies contaminants by comparing the presence or absence of each ASV in true positive samples (soil) with those in negative controls (nuclease‐free water), with contaminants expected to occur more frequently in the control samples (Davis et al. 2018). Eleven bacterial ASVs (614 reads) and 19 fungal ASVs (2939 reads) were identified as likely contaminants and removed from their respective datasets before further analysis. Taxon‐by‐sample abundance tables were created at the genus taxonomic level to allow for direct comparison with shotgun metagenome taxonomic classifications.

Functional inference of the bacterial communities was performed using Phylogenetic Investigation of Communities by Reconstruction of Unobserved States 2 (PICRUSt2) v2.6.1 (Douglas et al. 2020) to predict the functional capabilities and abundance of the identified communities based on marker gene sequences. Predicted functions were compared with those directly observed in the shotgun metagenome dataset to evaluate the accuracy of this approach. This assessment also provides a basis for considering the use of PICRUSt2 as a complementary, lower‐cost method for functional monitoring in kauri forests. The 16S ASV dataset was filtered to remove ASVs present in less than 10% of samples before running PICRUSt2. The Nearest Sequenced Taxon Index (NSTI) score was used to assess the accuracy of the predictions, with any ASV with an NSTI score > 2 removed from the analysis (Langille et al. 2013). The final predicted metagenome Kyoto Encyclopedia of Genes and Genomes (KEGG) Orthology (KO) abundance data were converted to relative abundances per sample.

#### 2.4.2 Shotgun Metagenome Data

Raw and demultiplexed metagenome sequencing reads were quality checked using the FastQC (https://www.bioinformatics.babraham.ac.uk/projects/fastqc/) and MultiQC tools (Ewels et al. 2016). Reads were trimmed using the BBDuk script via BBTools v39.01 (Bushnell 2017) to remove adapters, poor quality sequences, and PhiX reads (qtrim = rl, trimq = 25, *k* = 25, hdist = 1). Following trimming, non‐target DNA was removed in several stages. Human DNA was removed using the BBMap script via BBTools using the masked human reference genome, hg19 (minid = 0.95, maxindel = 3, bwr = 0.16, bw = 12, quickmatch, fast, minhits = 2, qtrim = rl, trimq = 10, untrim). Non‐target plant and animal DNA were removed using the KrakenTools v1.2 extract_kraken_reads.py (Lu et al. 2022) script against the inbuilt RefSeq database for plants, from Kraken v2.1.2 (Wood et al. 2019), and eight custom databases for animalia DNA built from NCBI taxonomies: annelida, arthropoda, chordata, mollusca, nematoda, platyhelminthes, tardigrada, and oomycota. Summary statistics of sample reads at each quality control stage were generated using SeqKit v2.4.0 (Shen et al. 2024), and the full dataset of read counts and quality metrics is available in Supporting Information S1.

After quality control of the metagenome reads, samples were assembled individually using MEGAHIT v1.2.9 (Li et al. 2016) with standard parameters and a minimum contig length of 1000 bp. Prokaryotic gene prediction was performed using Prodigal v2.6.3 (Hyatt et al. 2010) using standard parameters. Predicted genes were clustered at 95% sequence identity using CD‐HIT‐EST v4.8.1 (Fu et al. 2012) (parameters: –aS 0.9, −G 0, −g 1, −d 0) to generate a non‐redundant gene catalogue. Gene annotation was performed on the non‐redundant gene catalogue using eggNOG‐mapper v2.1.12 (Huerta‐Cepas et al. 2019) with DIAMOND alignment (Buchfink et al. 2021) against the eggNOG database v5.0. Coverage information of genes was determined using Bowtie2 v2.4.5 (−200, −maxins 800, −sensitive) (Langmead and Salzberg 2012) and CoverM v0.7.0 (contig, −m count) (Aroney et al. 2025). Raw gene counts were normalised by predicted gene length to account for differences in gene length.

To profile the taxonomic community of the shotgun metagenome reads, Kraken2 v2.1.2 (Wood et al. 2019) was used with the standard bacterial and fungal Kraken databases. Bracken v2.7 (Lu et al. 2017) was then used to generate the final abundance profiles of the metagenome sequences. Taxonomic lineage information was added to the profile using bit v1.9.21 (Lee 2022) and TaxonKit (Shen and Ren 2021).

To determine the functional potential of the soil bacterial communities, 10,866 unique KO numbers were extracted from the eggNOG annotation output. Database entries of these unique KOs were obtained from the KEGG database using kegg_pull v3.1.0 (Huckvale and Moseley 2023), which obtained information on 10,805 KOs. The KOs that did not have any information on KEGG are likely deprecated KOs. From the pulled entries, KEGG BRITE hierarchy information was extracted to understand the broad functional categories of genes. KEGG pathway information was extracted to understand the soil's bacterial communities' metabolic and nutrient cycling capabilities. KEGG pathways associated with “Organismal systems” and “Human disease” were removed from the dataset.

### 2.5 Statistical Analysis

All statistical analyses used R v4.4.0 (R Core Team 2021). To explore the taxonomic composition of the microbial communities, relative abundance counts (number of reads) were calculated at both the phylum and genus levels using the ‘phyloseq’ package v1.48.0 (McMurdie and Holmes 2013). Filtering was conducted to retain phyla with a mean relative abundance (MRA) > 1% across all samples and genera with a mean relative abundance > 0.5%. The resulting subset of taxa was used to visualise differences in microbial community composition at the phylum and genus levels across samples. Heatmaps were generated using the *plot_heatmap* function from the ‘phyloseq’ package with sample clustering based on Bray–Curtis dissimilarity and NMDS ordination of the community composition. Samples were grouped by their detection status of *P. agathidicida* as determined by LAMP results.

To identify genera with differential abundance between samples with and without *P. agathidicida* detection, differential abundance testing was performed using the *ancombc2* function from the ‘ANCOMBC’ package v2.6.0 (Lin and Peddada 2024). The analysis used default parameters, including sensitivity testing based on pseudo‐count addition, and adjusted *p*‐values using Holm‐Bonferroni correction. In this step, each taxon's differential abundance status was tested for consistency when a pseudo‐count was added to the zero counts of each taxon. Taxa that retained their significance status (*p*‐value remaining significant or non‐significant) were considered robust to the pseudo‐count addition and passed the sensitivity testing.

Rarefaction curves were generated using the *amp_rarecurve* function from the ‘ampvis2’ package v2.8.9 (Andersen et al. 2018) for ASVs (amplicon) and species (shotgun metagenome) to assess the species diversity across samples of varying sequencing depths. Sample curves plateaued, indicating that sequencing depth was sufficient for alpha diversity analysis (Figure S2). Samples were rarefied using the *rarefy_even_depth* function from the ‘phyloseq’ package (Reads per sample: amplicon 16S: 15,420, amplicon ITS: 4192, shotgun metagenome bacteria: 9,963,524, shotgun metagenome fungi: 42,440) before estimating alpha diversity at the genus level using observed richness and Shannon diversity index. The difference in diversity between the two detection groups was tested for significance using the Wilcoxon test.

To assess the beta diversity differences in the bacterial and fungal community composition between communities for both the amplicon and shotgun metagenome datasets, the raw counts of unique genera were first normalised using cumulative sum scaling (CSS) using the *cumNorm* function from the ‘metagenomeSeq’ package v1.46.0 (Paulson, Olson, et al. 2013; Paulson, Stine, et al. 2013) before creating a Bray–Curtis dissimilarity matrix using the *vegdist* function from ‘vegan’ v2.6.10 (Oksanen et al. 2020) and visualising using a principal coordinate analysis (PCoA). Significant differences between community composition were tested using permutational multivariate analysis of variance (PERMANOVA) using the *adonis2* function from the ‘vegan’ package. Procrustes rotation analysis was used to assess the congruence between the amplicon and metagenome ordinations for fungal community composition and bacterial community and functional composition using the *procrustes* function of the ‘vegan’ package. Permutational significance tests were conducted using the *protest* function with 999 permutations. The goodness of fit of the Procrustes analysis is measured by the *M* ^2^ statistic, representing the sum of squared differences between corresponding points of the two configurations. A lower *M* ^2^ indicates a closer match between configurations, while higher values indicate greater dissimilarity.

To explore the functional potential of the microbial community, relative abundance counts of KOs grouped at BRITE hierarchy levels 1 and 2 and KEGG pathway levels 2 and 3 were visualised using the ‘ComplexHeatmap’ R package v2.21.2 (Gu 2022). Default hierarchical clustering was used to order rows and columns of the heatmaps. To assess the consistency of KOs detected between amplicon‐based functional inference and shotgun metagenomic sequencing, we used the ‘ggVennDiagram’ package v1.5.2 (Gao et al. 2024). Differential abundance testing of KOs between detection status groups (detected vs. not detected) was calculated using the *ancombc2* function from the ‘ANCOMBC’ package. Only KOs passing the sensitivity testing were considered differentially abundant. Alpha diversity was estimated on KO counts rarefied using the *rrarefy* function of the ‘vegan’ package to 11,507,418 reads per sample for the shotgun dataset and 21,404,604 predicted gene copies per sample for the amplicon dataset. Observed richness was calculated using the *specnumber* function of the ‘vegan’ package, and differences between detection groups were tested using the Wilcoxon test. Functional composition between samples was assessed using CSS normalised KO counts to generate a Bray‐Curtis distance matrix using the *vegdist* function from ‘vegan’ before visualising using PCoA. Similarity between the shotgun metagenome and amplicon‐inferred functional prediction ordinations was assessed using Procrustes rotation analysis via the *procrustes* function of the ‘vegan’ package; permutational significance tests were conducted using the *protest* function with 999 permutations.

## 3 Results

### 3.1 Detection of *P. agathidicida*

#### 3.1.1 LAMP Analysis

*P. agathidicida* was detected in 39 out of 60 soil samples using the LAMP assay (Figure S3). Detection of the pathogen did not align well with canopy scores, as some trees with low canopy scores (indicating healthy trees) still detected *P. agathidicida*. All 21 soil samples where *P. agathidicida* was not detected were collected around trees with canopy scores of ≤ 3.5.

#### 3.1.2 Amplicon Sequencing

We explored whether *P. agathidicida* could be detected from the ITS1 amplicon dataset, which was primarily generated to characterise the wider fungal community. A BLASTn search of ASVs against reference *P. agathidicida* ITS sequences returned no matches, and taxonomic classification using the UNITE “all‐eukaryotes” database v8.3 similarly did not identify any sequences belonging to the *Phytophthora* genus. Examination of the primer‐template alignment showed that both ITS1‐F and ITS2 primers only partially matched the *P. agathidicida* reference sequence. The ITS1‐F primer aligned to a short region spanning 13 of 22 bases (100% identity, *E* = 1.85 × 10^−4^) within the 18S rRNA gene but did not show full‐length binding and lacked complementarity at the 3′ end of the primer. The ITS2 primer showed greater sequence similarity, aligning 16 of 20 bases (100% identity; *E* = 2.80 × 10^−6^) within the 5.8S rRNA gene; however, mismatches were also present at the 3′ end. The lack of full‐length primer binding and poor 3′‐end complementarity likely limited the ability of this primer set to amplify *P. agathidicida* ITS sequences.

#### 3.1.3 Shotgun Metagenome Sequencing

Across all shotgun metagenome samples, the proportion of reads classified by Kraken2 as *P. agathidicida* was very low, with most samples containing less than 0.002% of total reads (Figure S4). This indicates a low abundance *Phytophthora* signal, including reads classified as *P. agathidicida*, across these soils. One sample showed a notably higher number of reads classified as *P. agathidicida*: 2454 reads, 0.006% of total reads. This same sample also tested positive for *P. agathidicida* using LAMP analysis, representing a rare co‐occurrence between LAMP positive status and reads classified as *P. agathidicida* in this dataset. However, despite this isolated match, there was generally a poor alignment between the LAMP results and the shotgun metagenomic‐based detection via Kraken2 and Bracken analysis. In addition to reads classified as *P. agathidicida*, Kraken2 assigned reads across multiple other *Phytophthora* species in all samples. Given the short‐read length (~150 bp) and the use of *k*‐mer‐based classification, these assignments are interpreted as evidence of a low‐abundance, genus‐level *Phytophthora* signal rather than confirmation of multiple *Phytophthora* species in individual samples.

### 3.2 Microbial Community Taxonomic Composition

#### 3.2.1 General Characteristics of Amplicon and Shotgun Metagenome Datasets

The amplicon datasets obtained 2,415,068 bacterial reads and 1,108,149 fungal reads from the soil samples around kauri trees. The bacterial reads were classified into 19,020 ASVs, comprising 39 phyla, 253 families, and 447 genera. Fungal reads were classified into 7654 ASVs, comprising 10 phyla, 226 families, and 450 genera.

For the shotgun datasets, 7,230,854,958 sequences were obtained after quality control. From these, 871,250,634 reads were classified as bacterial, and 4,065,980 reads were classified as fungal. Bacterial reads were classified into 49 phyla, 562 families, and 1983 genera, and fungal reads into three phyla, 30 families, and 54 genera.

#### 3.2.2 Soil Microbial Community Composition

To investigate whether the presence of *P. agathidicida* is associated with microbial taxonomic composition, the relative abundance of bacterial phyla (> 1% MRA) and genera (> 0.5% MRA) between soils where *P. agathidicida* was detected and not detected (via LAMP analysis) using both amplicon and shotgun metagenome sequencing data was assessed. In the amplicon dataset, dominant phyla included Planctomycetota (35% MRA), Verrucomicrobiota (33% MRA), and Proteobacteria (13% MRA), which were dominant across both *P. agathidicida* detected and not detected soil samples (Figure S5A,B). In the shotgun metagenome dataset, samples predominantly comprised two phyla, Proteobacteria (57% MRA) and Actinobacteriota (34% MRA), while other phyla were present at much lower relative abundances. These dominant phyla appear consistent across samples where *P. agathidicida* was detected and not detected. At the genus level, both amplicon and shotgun metagenome datasets show similar profiles across both detection groups (Figure S5C,D). However, different genera were identified between the different sequencing methods, with the shotgun metagenome dataset providing a greater resolution and detecting a wider array of low‐abundance genera.

For the fungal dataset, relative abundance patterns at both the phylum and genus levels were broadly similar across *P. agathidicida* detection groups for both sequencing methods.

Ascomycota was the dominant phylum in the amplicon data set (58% MRA; Figure S6A); however, some samples exhibited a relatively higher abundance of Basidiomycota, corresponding to a decrease in Ascomycota abundance in those cases. The amplicon dataset detected a range of fungal genera, generally at low relative abundance (Figure S6C). Among these, *Mortierella* consistently showed the highest relative abundance across most samples (27% MRA). For the shotgun metagenome dataset, only 0.06% of total sequences were taxonomically categorised by the Kraken2 fungal database, suggesting a potential annotation bias and underestimation of fungal communities, with only three phyla, 30 families, and 54 genera detected. However, Ascomycota was identified as the dominant phylum (90% MRA), consistent with the amplicon dataset (Figure S6B). The genera *Fusarium* (9% MRA), *Thermothielavioides* (9% MRA), and *Colletotrichum* (8% MRA) were generally the most abundant across all samples in the shotgun metagenome dataset (Figure S6D).

Beta diversity analysis also showed no significant differences in microbial community composition between *P. agathidicida* detected and not detected soils. Visualisation using PCoA based on Bray–Curtis dissimilarity did not reveal any clear groupings by *P. agathidicida* detection status (Figure S7). This was supported by PERMANOVA results, which indicated no statistically significant variation between groups (*p* > 0.05). PERMANOVA revealed significant differences in soil microbial community composition among sites (Cascades, Piha, and Huia; *p* < 0.05), but further investigation was beyond the scope of this study and will be addressed in future work.

**FIGURE 1:**
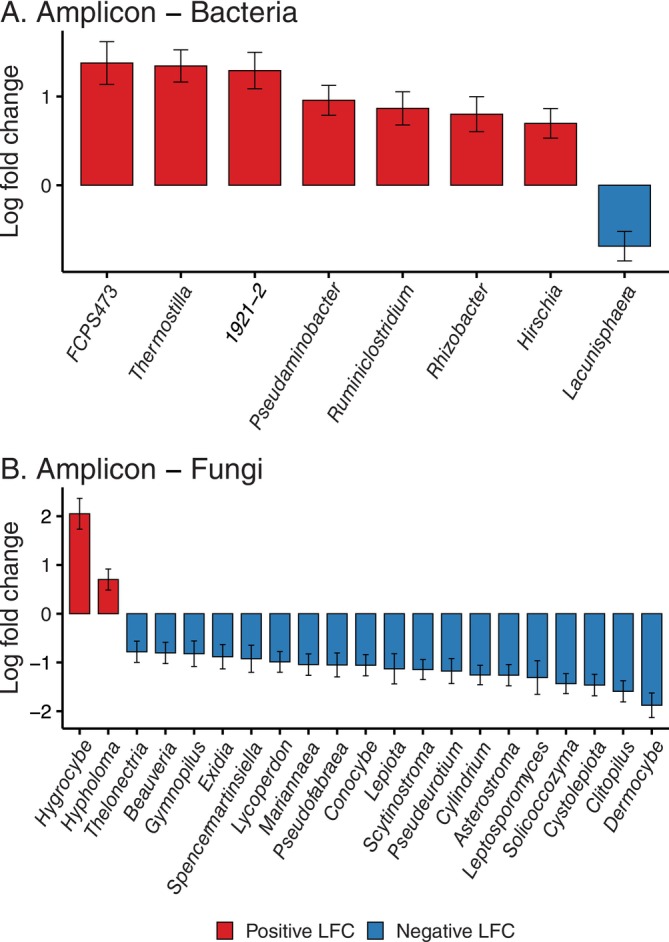
Bacterial (A) and fungal (B) genera showing differential abundance between soil samples where *P. agathidicida* was detected and not detected (adjusted *p* < 0.05 following Holm‐Bonferroni correction). Genera with a positive log fold change (natural log) show increased abundance in soils without *P. agathidicida* detected and genera with a negative log fold change (natural log) show increased abundance in soils with *P. agathidicida* detected. LFC, log fold change.

Differential abundance analysis of the amplicon dataset revealed several bacterial and fungal genera that varied significantly between soils where *P. agathidicida* was detected against those where they were not (Figure 1; adjusted *p* < 0.05 following Holm‐Bonferroni correction). Among bacteria, *Lacunisphaera* (natural log fold change [LFC] −0.69) was less abundant in soils where *P. agathidicida* was undetected, whereas *FCPS473* (LFC 1.38), *Thermostilla* (LFC 1.34), *1921–2* (LFC 1.29), *Pseudaminobacter* (LFC 0.96), *Ruminiclostridium* (LFC 0.87), *Rhizobacter* (LFC 0.80), and *Hirschia* (LFC 0.70) were more abundant; however, none of these genera passed the sensitivity testing (pseudo count addition to zero count taxa). For fungi, *Hygrocybe* (LFC 2.05) and *Hypholoma* (LFC 0.70) were enriched in soils where *P. agathidicida* was not detected, with 19 genera more abundant in soils where the pathogen was present. Among these fungal genera, only three taxa passed the sensitivity testing: *Mariannaea* (LFC‐1.04), *Pseudofabraea* (LFC‐1.05), and *Pseudeurotium* (LFC‐1.18). In both bacterial and fungal communities, the differentially abundant taxa were of low relative abundance across individual samples (< 1%), indicating that these differences represent subtle shifts in community composition rather than dominant taxa driving overall structure. No differentially abundant genera were identified in the shotgun metagenome datasets for either bacteria or fungi.

#### 3.2.3 Microbial Community Diversity

There were no significant differences in alpha diversity estimates between samples with and without *P. agathidicida* detection. This was consistent across both observed richness and Shannon diversity estimates (*p* > 0.05) for bacterial and fungal genera, based on both amplicon and shotgun metagenome datasets (Figure S8).

Comparisons between the sequencing methods revealed some differences in richness estimates. Shotgun metagenome sequencing estimated a higher bacterial observed richness compared to amplicon sequencing. In contrast, fungal genera showed a higher observed richness in the amplicon dataset, with shotgun metagenome sequencing recovering relatively few fungal genera (Figure S8D), likely due to the reference database used for taxonomic classification.

### 3.3 Functional Potential of Bacterial Communities

Functional potential of the bacterial communities was assessed by comparing KOs inferred by PICRUSt2 (from amplicon data) and genes predicted and annotated using Prodigal and eggNOG (from shotgun metagenome data). PICRUSt2 inferred 7518 unique KOs from 2183 ASVs, with an average NSTI value of 0.156, indicating a fair match between sequenced ASVs and the reference genomes. For the shotgun metagenome dataset, 8,519,250 genes were predicted; 30% of the predicted genes had an associated KO number, with 10,866 unique KOs identified across all samples.

The predicted KOs were grouped into higher‐level functional categories using BRITE and KEGG pathway hierarchies. Despite differences in sequencing methodology and KO inference/prediction, the functional profiles generated from shotgun (Figure S9) and amplicon data (Figure S10) were relatively consistent. Across both datasets, the most abundant category was the BRITE level 3 category “Transporters,” a group of proteins involved in cellular import and export processes. Similarly, when KOs were grouped into KEGG pathways, both datasets revealed “Carbohydrate metabolism” and “Amino acid metabolism” as the most prominent functional pathways, highlighting the high microbial activity associated with nutrient cycling and organic matter processing.

Samples grouped based on LAMP‐based detection of *P. agathidicida* showed no major differences in KO‐level relative abundances between soils where *P. agathidicida* was detected and not detected. The consistent patterns of functional abundance across detection groups indicate that the presence of the pathogen may not be associated with large‐scale shifts in functional potential detectable at broad KEGG category levels.

Differential abundance testing of individual KOs revealed no significantly differentially abundant KOs in the shotgun metagenome dataset when comparing samples where *P. agathidicida* was detected and not detected. In contrast, exploratory analysis of PICRUSt2‐inferred KOs identified four putative differences; however, given the lack of support in the shotgun dataset, these should be interpreted cautiously.

While no significant differences in functional alpha diversity (based on observed richness) were observed between *P. agathidicida*‐detected and not detected soils for either sequencing method (*p* > 0.05, Figure S11), no clustering by *P. agathidicida* detection was observed using PCoA (Figure S12); PERMANOVA confirmed no significant differences (PICRUSt2: *R* ^2^ = 0.005, *p* = 0.83, *n* = 58. eggNOG: *R* ^2^ = 0.021, *p* = 0.271, *n* = 60) in KO composition between detection groups. PERMANOVA analysis indicated significant differences in the functional profiles of soil microbial communities across sites (*p* < 0.05); however, these patterns were not examined in detail here as they are beyond the scope of this study and will be addressed in future work.

#### 3.3.1 Concordance Between Amplicon and Shotgun Metagenome Datasets

**FIGURE 2:**
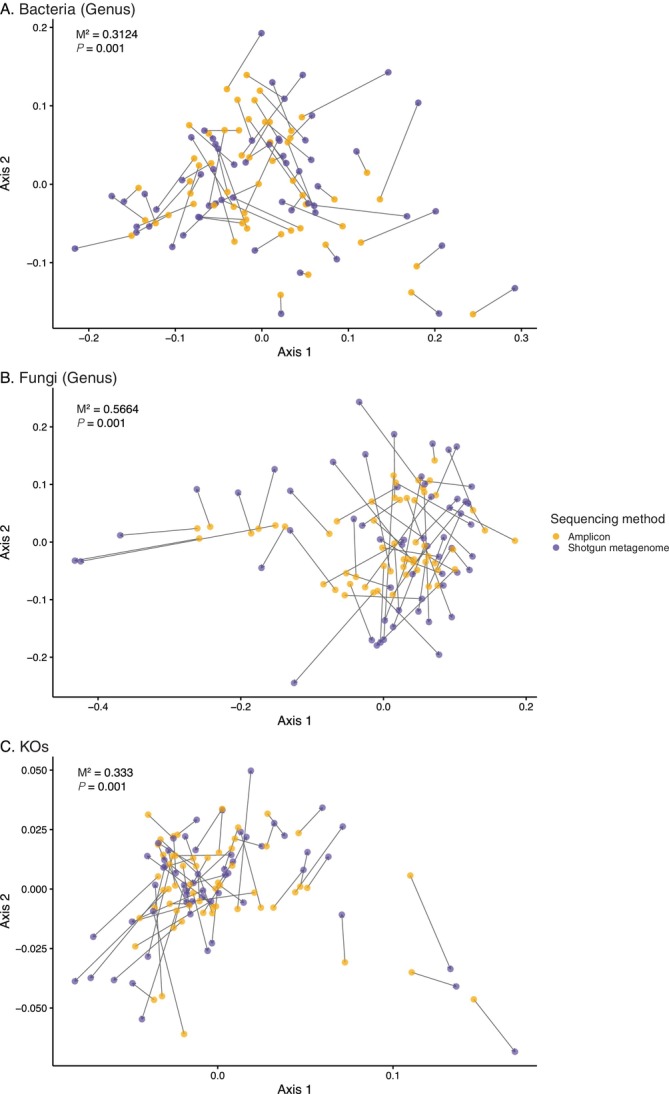
Procrustes analysis comparing community composition profiles of (A) bacterial genera, (B) fungal genera, and (C) KEGG Orthologs (KO) derived from amplicon (gold) and shotgun metagenome (purple) sequencing. Arrows connect matching samples, with arrow length indicating the degree of dissimilarity between the two datasets for that sample (longer arrows = greater discordance). For each rotation, the *M* ^2^ statistic and associated *p*‐value are shown, where *M* ^2^ represents the proportion of variance unexplained by the Procrustes fit (lower values indicate better agreement between datasets).

Procrustes analysis was used to assess the similarity in variation among samples of bacterial and fungal genus‐level community composition derived from amplicon and shotgun metagenome data (Figure 2A,B). For bacterial communities, a moderate alignment was observed (*M* ^2^ = 0.3124, *p* = 0.001), suggesting a considerable agreement between sequencing methods despite differences in the taxa detected. For the fungal communities, the alignment between the sequencing methods was weaker (*M* ^2^ = 0.5664, *p* = 0.001) due to the higher *M* ^2^ value indicating greater dissimilarity between the amplicon and shotgun datasets. The Procrustes test used to compare community functional profiles between shotgun and amplicon‐inferred KO predictions (Figure 2C) showed a moderate alignment between sequencing methods (*M* ^2^ = 0.333, *p* = 0.001).

Comparison of the KOs annotated by amplicon‐based inference and shotgun metagenomic sequencing showed that 6972 KOs were shared between both methods (Figure S13). The 546 KOs only identified in the amplicon‐inferred dataset are likely false positives, as they were not detected through direct sequencing of the environmental DNA (shotgun metagenomics). Included in these is K06420, which was the only differentially abundant KO showing increased abundance in soils where *P. agathidicida* was not detected. The 3833 KOs absent from the amplicon‐based inference likely reflect false negatives and highlight the limitations of the PICRUSt2 method.

## 4 Discussion

### 4.1 Summary of Key Findings

Our study addressed four key questions to assess the methods used to detect and measure the relationship between the structure and function of soil microbial communities surrounding kauri and the presence of *P. agathidicida*. Firstly, in comparing detection methods, LAMP analysis provided both detection and viability information of the pathogen, whereas amplicon sequencing failed to detect any reads assigned to the *Phytophthora* genus. In contrast, shotgun metagenome sequencing detected *P. agathidicida*‐associated DNA in all soil samples, although at low abundance. Secondly, observing the microbial community structure regarding taxonomic diversity and composition, we found that it was not substantially affected by *P. agathidicida* detection; however, several microbial taxa were differentially abundant between samples where the pathogen was detected and those where it was not. Similarly, analysis of community functional potential showed limited differentiation between samples with and without pathogen detection. Finally, shotgun metagenomic and amplicon sequencing approaches concord strongly in their broad‐level taxonomic and functional profiles. Collectively, these findings suggest that while *P. agathidicida* may influence the abundance of specific microbial taxa, its detection is not strongly associated with shifts in the surrounding soil microbial community's core taxonomic or functional structure.

### 4.2 *P. agathidicida* Detection Across Different Methods

This study compared three methods for detecting *P. agathidicida* in soil samples collected around kauri trees: LAMP analysis combining traditional baiting techniques with rapid amplification, and two sequencing‐based methods: amplicon sequencing of the ITS1 region and shotgun metagenomic sequencing. Together, these methods offer a range of sensitivity, specificity, and insight into pathogen detection and broader microbial context.

Amplicon sequencing of the ITS1 region failed to detect *P. agathidicida* or assign any ASVs to the Oomycota class, despite its detection being confirmed through shotgun metagenomics. This likely reflects both the low abundance of the pathogen in the soil and the taxonomic bias of ITS1 primers, which are optimised for capturing broad fungal diversity and, therefore, may not efficiently amplify oomycetes, especially in environments that are dominated by other fungal species (Sapkota and Nicolaisen 2015; Schoch et al. 2012). Previous studies have demonstrated greater success with *P. agathidicida* detection using more targeted PCR assays, such as nested PCR and more targeted primer sets for *Phytophthora* species or oomycete detection (Hunter et al. 2024; Legeay et al. 2019; Palmer et al. 2025). To reliably profile fungal and oomycete or *Phytophthora* communities using amplicon sequencing, primer sets must be specifically designed to capture both groups, or separate amplification steps should be undertaken for each target community.

Using shotgun metagenomics, Kraken2 detected *Phytophthora*‐associated reads across all samples, indicating the presence of a low‐abundance *Phytophthora* signal in these soils. The consistent low abundance of *P. agathidicida* suggests that shotgun metagenomic sequencing is sensitive to background *Phytophthora* DNA even when reads classified as the target pathogen occur at very low levels.

Although shotgun metagenomics was sufficiently sensitive to capture low‐abundance *Phytophthora*‐associated DNA, this approach cannot distinguish DNA derived from active infections from residual or extracellular DNA, limiting its utility for diagnosing active disease. In contrast, LAMP‐based detection provides targeted, species‐specific detection and remains the most reliable indicator of *P. agathidicida* presence and viability in these samples. As a result, shotgun metagenomic detection showed limited alignment with LAMP‐based results but instead offers complementary insight into the broader microbial and oomycete community associated with kauri soils, without the primer biases associated with targeted amplicon approaches.

In addition to reads classified as *P. agathidicida*, shotgun metagenomic data included reads assigned to multiple other *Phytophthora* species, consistent with previous reports of diverse *Phytophthora* communities co‐occurring in kauri forest soils (Beever and Bellgard 2010; Hunter et al. 2024; Waipara et al. 2013). Together, this indicates that shotgun metagenomics primarily captures a low‐abundance, genus‐wide *Phytophthora* signal in soil. However, this signal should be interpreted cautiously due to the limited taxonomic resolution of the shotgun metagenomic approach used. Taxonomic classification was based on short shotgun metagenomic reads (~150 bp) and a *k*‐mer‐based classifier (Kraken2), which has limited discriminatory power at the species level, particularly for closely related taxa (Govender and Eyre 2022). As such, species‐level assignments, including those classified as *P. agathidicida*, are best viewed as reflecting genus‐level *Phytophthora* signal across all samples rather than definitive species detection.

The three approaches used in this study provide complementary perspectives on the detection of *P. agathidicida*. The LAMP‐based detection yielded species‐specific identification, which is more closely aligned with targeted surveillance needs, whereas shotgun metagenomics detected low‐abundance reads taxonomically assigned to *Phytophthora* across all samples, reflecting the high sensitivity of this approach in tracing environmental DNA. The widespread detection of low‐abundance *Phytophthora*‐associated DNA by shotgun metagenomics likely reflects background environmental signal and the persistence of DNA in soils, rather than definitive evidence of an active infection. Although amplicon sequencing of the ITS1 region failed to detect *P. agathidicida* directly, likely due to primer bias and low DNA abundance, it still revealed broader community profiles consistent with shotgun metagenome data at higher taxonomic levels. Together, these results highlight that discrepancies among methods are driven by fundamental differences in sensitivity, specificity, and biological interpretation, underscoring the strengths and limitations of each method depending on the research goal, whether it be presence/absence, viability, or broader ecological context.

### 4.3 Impacts of *P. agathidicida* Presence on Microbial Community Composition

Although the overall microbial community composition and diversity were not significantly different between soil samples where *P. agathidicida* was detected and not detected, our results revealed a subtle, but significant shift in the abundance of specific microbial taxa that may be responding to the presence or absence of the pathogen. The differences in abundance of these taxa did not create large‐scale shifts in the community composition. However, they may still reflect relevant responses to the presence or absence of the pathogen. The bacterial genera *Rhizobacter* and *Hirschia* were significantly higher in abundance in soils without *P. agathidicida* detection. These taxa have been previously associated with disease suppression and observed to be higher in soils around healthy plants (Ketehouli et al. 2024; Siegel‐Hertz et al. 2018; Wright et al. 2022). *Rhizobacter* species, in particular, are recognized as plant growth‐promoting bacteria and are also known to suppress the growth of pathogens by producing siderophores and secondary metabolites (Abbas et al. 2022).

Similarly, several of the fungal genera that had higher abundance in soils where *P. agathidicida* was detected have been linked with antagonistic activity towards plant pathogens. Members of the *Beauveria* genus, for example, are known to produce a wide array of enzymes and secondary metabolites and show antagonistic effects towards a range of phytopathogens, including *Phytophthora* species (Lozano‐Tovar et al. 2017, 2013; Pachoute et al. 2024). The *Gymnopilus* genus has also previously been linked with disease suppression, showing antagonistic effects towards bacterial and fungal pathogens (Ranadive et al. 2013). Although these taxa did not drive large shifts in overall community structure, their differential abundance suggests potential direct or indirect interactions with *P. agathidicida*. It may reflect underlying microbial dynamics related to disease expression.

### 4.4 Functional Shifts in the Microbial Community

Although several bacterial taxa were identified as differentially abundant, linking these taxa to specific metabolic pathways was not supported by gene‐level evidence. Differential abundance analysis of individual KOs identified no significant differences in the shotgun metagenomic dataset. While a small number of KO‐level differences were inferred from amplicon‐based functional predictions, these signals were not supported by the shotgun metagenome dataset and were therefore interpreted as likely artifacts of predictive inference rather than biologically meaningful functional differences (Sun et al. 2020). At broader functional scales, the predicted functional profiles remained relatively consistent. This consistency, especially at higher functional categorical levels (KEGG pathway levels 2 and 3 and BRITE hierarchy levels 1 and 2), suggests the presence of functional redundancy within the microbial community. In such environments, multiple microbial taxa may carry out similar ecological functions, allowing the broader functional profile of the community to remain stable even when its taxonomic composition shifts (Li et al. 2021; Nannipieri et al. 2017). This redundancy may buffer the broad functions of a community against environmental disturbances, such as pathogen invasion, and supports the idea that soil microbial communities maintain key ecosystem processes through overlapping metabolic capacities (Chen et al. 2022; Gao et al. 2021). Furthermore, it is possible that the presence of *P. agathidicida*, which likely comprises a relatively small proportion of total microbial biomass or exists in a metabolically inactive state, does not exert a strong effect to shift microbial community function at the scale captured by higher‐level KEGG‐level analyses. The localized microbial response to the pathogen may also be spatially restricted or masked by larger‐scale variations in soil properties.

### 4.5 Alignment Between Shotgun Metagenome and Amplicon‐Derived Profiles

While specific bacterial and fungal phyla were consistently identified in both amplicon and shotgun sequencing datasets, the genus‐level profiles differed. These discrepancies likely reflect differences in detection sensitivity, sequencing depth, and database completeness between the two methods. The shotgun metagenome dataset, which had a much greater sequencing depth, revealed a broader diversity of bacterial genera and likely recovered more rare or low‐abundance taxa (Brumfield et al. 2020; Madison et al. 2023). However, for fungal taxa, taxonomic resolution was limited. Although shotgun sequencing has the potential to detect a broader array of fungal diversity, this analysis was restricted by the limited reference database used (Kraken2), which included only 455 fungal species. Use of this database likely underestimated the true fungal diversity in the soil and emphasizes the importance of database selection and the need for improved fungal reference databases to fully utilise shotgun metagenome data derived from diverse soil ecosystems (Avershina et al. 2025).

Despite differences in taxonomic composition between sequencing methods, the predicted functional profiles of the microbial communities remained relatively consistent. The PICRUSt2‐inferred KOs and shotgun metagenome‐derived annotations (via eggNOG) exhibit fundamental differences in their approaches to classifying genes and KOs. PICRUSt2 relies on phylogenetic inference from 16S rRNA gene sequences, whereas the shotgun metagenomic approach provides direct evidence of functional genes present in the community (Bharti and Grimm 2021; Douglas et al. 2020). Despite these differences in approach, the overall patterns of microbial community function were largely consistent in this study. The consistency between these two methods, especially at higher levels of functional groupings, suggests that at least at the broad scale, functional potential can be inferred from amplicon data, which is consistent with previous studies on soil environments (Toole et al. 2021). The accuracy of the PICRUSt2 predictions in this study was supported by a moderately low NSTI value, indicating a good alignment between observed ASVs and available reference genomes in the PICRUSt2 database (Douglas et al. 2020). However, the two methods had substantial differences in the total number of identified KOs. The large number of KOs uniquely identified by shotgun metagenome sequencing likely reflects the improved sensitivity and coverage of genes, especially for rare or uncharacterised functions.

These findings also underscore the advantages and disadvantages of each sequencing approach in terms of analytical resolution and study design. Amplicon‐based functional inference provided results that were largely consistent with shotgun sequencing at the level of broad functional categories, indicating that PICRUSt2 can be a reliable and cost‐effective approach for monitoring microbial functional potential in kauri forest soils. This makes amplicon sequencing a practical choice for long‐term or large‐scale studies where resources are limited. In contrast, shotgun metagenomics, while more resource‐intensive, offers additional advantages that cannot be obtained from amplicon sequencing. Beyond profiling community composition and function, shotgun data can be used to reconstruct metagenome‐assembled genomes, enabling strain‐level resolution and insights into novel or uncultured organisms. Importantly, shotgun sequencing provides an integrated view of bacterial, fungal, and oomycete communities within a single dataset, providing an integrated view of the pathogen and its surrounding microbiome. Together, these results suggest that amplicon sequencing with functional inference is sufficient for addressing many community‐level questions, whereas shotgun metagenomics is most appropriate for studies requiring genome recovery, cross‐domain integration, or fine‐scale resolution of microbial community structure.

## 5 Conclusion

Overall, our study aimed to evaluate how different molecular‐based tools compared in their ability to detect *P. agathidicida* and characterise the associated changes in soil microbial communities in soils surrounding kauri trees. We have provided a multifaceted view of how the presence of this plant pathogen may influence microbial diversity, composition, and function while also evaluating the strengths and limitations of the methods used.

Our findings demonstrate that no single method provides a complete picture of pathogen detection or microbial community composition. Although *P. agathidicida* detection was associated with subtle changes in microbial community composition, our findings suggest that direct microbial responses to the pathogen presence may not be the dominant driver of community structure. Instead, indirect effects, such as declining tree health associated with canopy loss and gummosis, may play a more substantial role in shaping the soil microbial community. Additionally, spatial variation and soil physicochemical properties require further investigation, as these broader environmental factors may influence how pathogen presence interacts with the soil microbial community structure and function.

Beyond methodological insights, these results have practical relevance for kauri dieback management. The enrichment of specific microbial taxa in the presence or absence of *P. agathidicida* highlights components of the soil microbiome that warrant further investigation for their potential role in disease suppression. From a management perspective, our findings support a tiered framework in which targeted assays such as LAMP are used for operation surveillance and decision‐making, while amplicon and shotgun metagenomic approaches provide broader ecological context to inform long‐term monitoring and research priorities.

Future studies should integrate both amplicon and shotgun metagenome sequencing across finer spatial gradients and couple this with environmental data to help reveal the biotic and abiotic factors involved in shaping forest soil microbiomes to develop a deeper understanding of how kauri dieback influences and is influenced by the belowground microbial community.

## Author Contributions

**Zoe King:** writing – original draft, writing – review and editing, data curation, formal analysis, visualisation, investigation, conceptualisation, software, methodology. **Hannah L. Buckley:** writing – review and editing, methodology, conceptualisation, supervision. **Gavin Lear:** writing – review and editing, conceptualisation, supervision. **Brent Seale:** writing – review and editing, supervision. **Kevin C. Lee:** writing – review and editing, methodology, supervision, software. **Luitgard Schwendenmann:** writing – review and editing, funding acquisition. **Donnabella C. Lacap‐Bugler:** funding acquisition, conceptualisation, supervision, writing – review and editing, project administration, resources.

## Funding

This work was supported by New Zealand's Biological Heritage (C09X1817).

## Conflicts of Interest

The authors declare no conflicts of interest.

## Supporting information


**Data S1:** emi470324‐sup‐0001‐File1.pdf.

**Table S1:** Amplicon samples with < 1000 reads that were removed prior to statistical analyses.
**Figure S1:** Map of the three sampling sites (Cascades, Piha, Huia) in the North Island, New Zealand, that each contain two plots (*n* = 6) where 16 kauri trees, per plot, were selected for soil sampling (*n* = 96).
**Figure S2:** Rarefaction curves showing observed microbial richness in amplicon and shotgun metagenome datasets. A, B Show rarefaction curves for bacterial (A) and fungal (C) ASVs from amplicon sequencing, while B, D show rarefaction curves for bacterial (B) and fungal (D) species identified from shotgun metagenome data. Each curve represents an individual soil samples collected from around kauri trees, coloured by *P. agathidicida* (PA) detection status based on LAMP analysis. The dotted line indicates the minimum sequencing depth to with each dataset was rarefied to.
**Figure S3:** Canopy healthy scores of kauri trees across sites (Cascades, Huia, and Piha, within the Waitākere Ranges, Auckland, New Zealand), with point size proportional to the number of trees at each site‐score combination. Each point is displayed as a pie chart showing the proportion of trees testing positive or negative for *P. agathidicida* by LAMP analysis.
**Figure S4:** Read count of *P. agathidicida*‐associated DNA per sample against detection status inferred by LAMP analysis (detected *n* = 39, not detected *n* = 21). Boxes represent the interquartile range of the data (25th and 75th percentiles), whiskers show the largest and smallest values 1.5× the IQR and median values are represented by the bar within each box.
**Figure S5:** Heatmap of the relative abundance of the top bacterial phyla (> 1% MRA), and genera (> 0.5% MRA) identified using amplicon and shotgun metagenomic sequencing. Detection of *P. agathidicida* was determined by LAMP analysis (detected *n* = 37 [amplicon], 39 [shotgun], or not detected [*n* = 21]). The heatmap uses NMDS ordination to order the samples (columns) arranging them based on their similarity in microbial community composition as captured by the first ordination axis.
**Figure S6:** Heatmap of the relative abundance of the top fungal phyla (> 1% MRA), and genera (> 0.5% MRA) identified using amplicon and shotgun metagenomic sequencing. Detection of *P. agathidicida* was determined by LAMP analysis (detected *n* = 38 (amplicon), 39 (shotgun), or not detected *n* = 19 (amplicon), 21 (shotgun)). The heatmap uses NMDS ordination to order the samples (columns) arranging them based on their similarity in microbial community composition as captured by the first ordination axis.
**Figure S7:** Principal Coordinates Analysis (PCoA) of bacterial (A, B) and fungal (C, D) genus‐level community composition of Bray‐Curtis distance matrices from amplicon and shotgun metagenome datasets. Samples were normalised by cumulative‐sum scaling. Points are coloured based on LAMP detection of *P. agathidicida* (Bacterial dataset: detected *n* = 37) (amplicon), 39 (shotgun) and not detected *n* = 21 (amplicon and shotgun). Fungal dataset: detected *n* = 38 (amplicon), 39 (shotgun), or not detected *n* = 19 (amplicon), 21 (shotgun).
**Figure S8:** Measures of bacterial (A, B) and fungal (C, D) taxonomic alpha diversity at the genus level estimated by Shannon diversity and observed richness from amplicon and shotgun metagenome datasets. Samples are grouped based on the LAMP detection of *P. agathidicida* (Bacterial dataset: detected *n* = 37 (amplicon), 39 (shotgun) and not detected *n* = 21 (amplicon and shotgun). Fungal dataset: detected *n* = 38 (amplicon), 39 (shotgun), or not detected *n* = 19 (amplicon), 21 (shotgun)). Samples were rarefied to an even depth (Reads per sample: amplicon 16S: 15,420, amplicon ITS: 4192, shotgun bacteria: 9,963,524, shotgun fungi: 42,440). Boxes represent the interquartile range of the data (25th and 75th percentiles), whiskers show the largest and smallest values 1.5× the IQR and median values are represented by the bar within each box.
**Figure S9:** Heatmaps showing the relative abundance of KOs identified through shotgun metagenome sequencing and eggNOG annotation, grouped by KEGG functional classifications and LAMP detection of *P. agathidicida* (detected *n* = 39, not detected *n* = 21). (A) KOs grouped according to KEGG BRITE hierarchies at level 2 and level 3, (B) KOs grouped by KEGG pathway levels 1 and level 2. Samples are clustered based on default Euclidian clustering from the ComplexHeatmap R package. Pathways associated with Organismal systems and Human disease were removed from the dataset.
**Figure S10:** Heatmaps showing the relative abundance of KOs identified through functional inference by PICRUSt2 of ASVs from amplicon sequencing, grouped by KEGG functional classifications and LAMP detection of *P. agathidicida* (detected *n* = 37, not detected *n* = 21). (A) KOs grouped according to KEGG BRITE hierarchies at level 2 and level 3, (B) KOs grouped by KEGG pathway levels 1 and level 2. Samples are clustered based on default Euclidian clustering from the ComplexHeatmap R package. Pathways associated with Organismal systems and Human disease were removed from the dataset.
**Figure S11:** Boxplots showing estimated alpha diversity of microbial functional potential based on KO profiles from (A) amplicon sequencing (via PICRUSt2) and (B) shotgun metagenome sequencing (via eggNOG annotation). Samples were grouped by LAMP detection of *P. agathidicida* (detected *n* = 37 (amplicon), 39 (shotgun), not detected *n* = 21 (amplicon), 21 (shotgun)). Boxes represent the interquartile range of the data (25th and 75th percentiles), whiskers show the largest and smallest values 1.5× the IQR and median values are represented by the bar within each box.
**Figure S12:** Principal Coordinates Analysis (PCoA) of amplicon‐based inference (PICRUSt2) and shotgun metagenome sequencing (eggNOG annotation) of KOs based on Bray‐Curtis distance matrices. Samples were normalised by CSS. Points are coloured based on LAMP detection of *P. agathidicida* (detected *n* = 37 (amplicon), 39 (shotgun), not detected *n* = 21 (amplicon), 21 (shotgun)).
**Figure S13:** Venn diagram showing the overlap of KOs identified from shotgun metagenome sequencing (eggNOG annotation) and amplicon‐based functional inference (PICRUSt2).

## Data Availability

All sequencing data generated in this study have been submitted to the EMBL European Nucleotide Archive under BioProject PRJEB96056. The data that support the findings of this study are available on request from the corresponding author. The data are not publicly available due to privacy or ethical restrictions.
