# Supplementary material for: Comparative Amplicon and Shotgun Metagenome Profiling of Soil Microbial Communities in Kauri Forests Affected by Phytophthora agathidicida
Source: Environ Microbiol Rep. 2026 Apr 1;18(2):e70324. doi: 10.1111/1758-2229.70324 (PMC13045465; doi:10.1111/1758-2229.70324)
Supplement: Supplementary file 2 — Table S1: Amplicon samples with < 1000 reads that were removed prior to statistical analyses. Figure S1: Map of the three sampling sites (Cascades, Piha, Huia) in the North Island, New Zealand, that each contain two plots (n = 6) where 16 kauri trees, per plot, were selected for soil sampling (n = 96). Figure S2: Rarefaction curves showing observed microbial richness in amplicon and shotgun metagenome datasets. A, B Show rarefaction curves for bacterial (A) and fungal (C) ASVs from amplicon sequencing, while B, D show rarefaction curves for bacterial (B) and fungal (D) species identified from shotgun metagenome data. Each curve represents an individual soil samples collected from around kauri trees, coloured by P. agathidicida (PA) detection status based on LAMP analysis. The dotted line indicates the minimum sequencing depth to with each dataset was rarefied to. Figure S3: Canopy healthy scores of kauri trees across sites (Cascades, Huia, and Piha, within the Waitākere Ranges, Auckland, New Zealand), with point size proportional to the number of trees at each site‐score combination. Each point is displayed as a pie chart showing the proportion of trees testing positive or negative for P. agathidicida by LAMP analysis. Figure S4: Read count of P. agathidicida‐associated DNA per sample against detection status inferred by LAMP analysis (detected n = 39, not detected n = 21). Boxes represent the interquartile range of the data (25th and 75th percentiles), whiskers show the largest and smallest values 1.5× the IQR and median values are represented by the bar within each box. Figure S5: Heatmap of the relative abundance of the top bacterial phyla (> 1% MRA), and genera (> 0.5% MRA) identified using amplicon and shotgun metagenomic sequencing. Detection of P. agathidicida was determined by LAMP analysis (detected n = 37 [amplicon], 39 [shotgun], or not detected [n = 21]). The heatmap uses NMDS ordination to order the samples (columns) arranging them based on their simila [file EMI4-18-e70324-s001.docx]

Table S1: Amplicon samples with <1000 reads that were removed prior to statistical analyses.

| **16S samples** | **ITS samples** |
| --- | --- |
| NRT-PI-K4-16S | NRT-CI-D26-ITS |
| NRT-HI-K1-16S | NRT-HI-K4-ITS |
|  | NRT-PU-D124-ITS |

Codes H, P, C = Sampling site (Huia, Piha, Cascades). Codes I and U = Sampling plot (I = Plot 1, U = Plot 2).


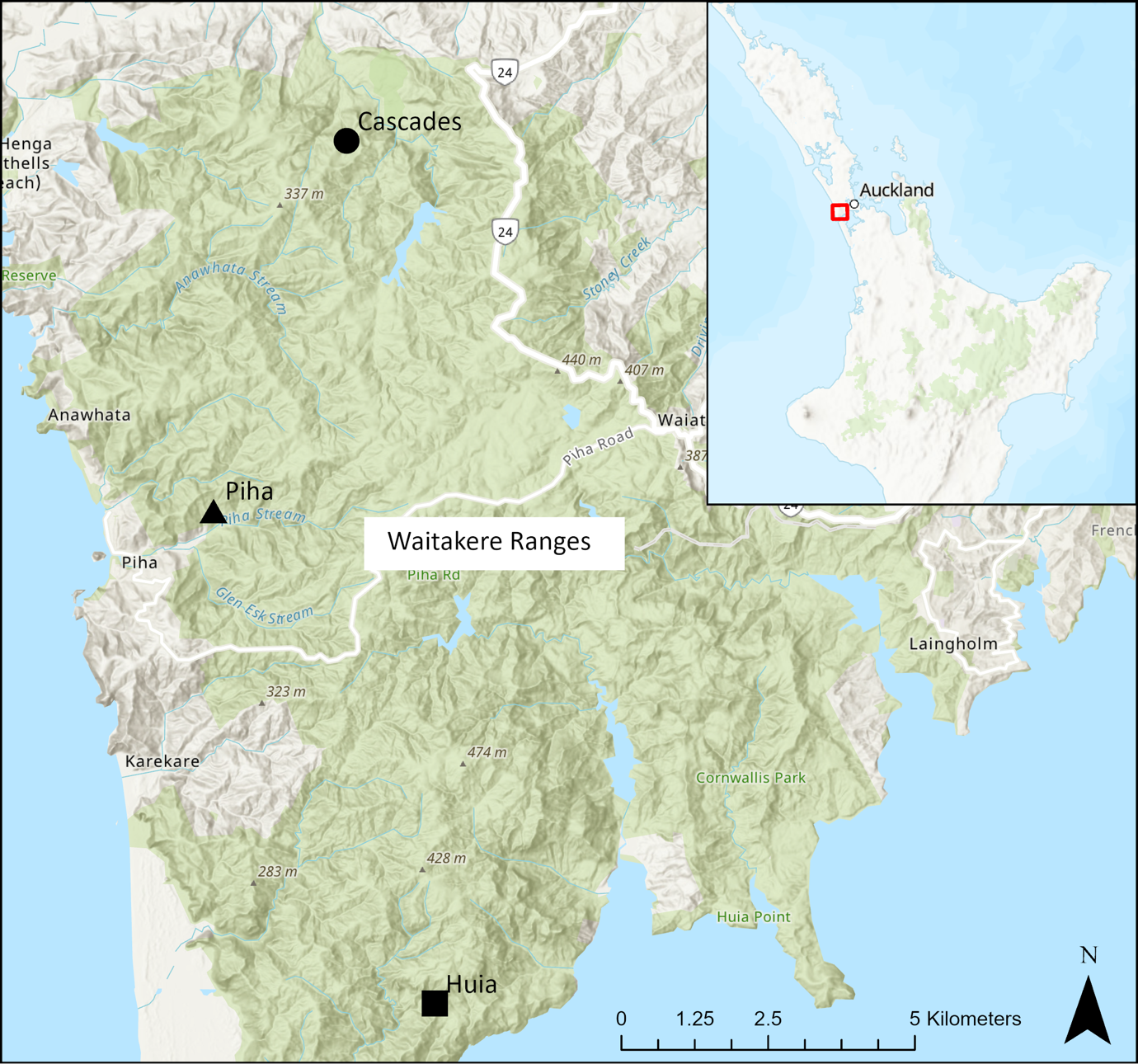


Figure S1: Map of the three sampling sites (Cascades, Piha, Huia) in the North Island, New Zealand, that each contain two plots (*n* = 6) where 16 kauri trees, per plot, were selected for soil sampling (*n* = 96).

Figure S2: Rarefaction curves showing observed microbial richness in amplicon and shotgun metagenome datasets. Figures A and B show rarefaction curves for bacterial (A) and fungal (C) ASVs from amplicon sequencing, while figures B and D show rarefaction curves for bacterial (B) and fungal (D) species identified from shotgun metagenome data. Each curve represents an individual soil samples collected from around kauri trees, coloured by *P. agathidicida* (PA) detection status based on LAMP analysis. The dotted line indicates the minimum sequencing depth to with each dataset was rarefied to.

Figure S3: Canopy healthy scores of kauri trees across sites (Cascades, Huia, and Piha, within the Waitākere Ranges, Auckland, New Zealand), with point size proportional to the number of trees at each site-score combination. Each point is displayed as a pie chart showing the proportion of trees testing positive or negative for *P. agathidicida* by LAMP analysis.

Figure S4: Read count of *P. agathidicida*-associated DNA per sample against detection status inferred by LAMP analysis (detected *n* = 39, not detected *n* = 21). Boxes represent the interquartile range of the data (25^th^ and 75^th^ percentiles), whiskers show the largest and smallest values 1.5x the IQR and median values are represented by the bar within each box.


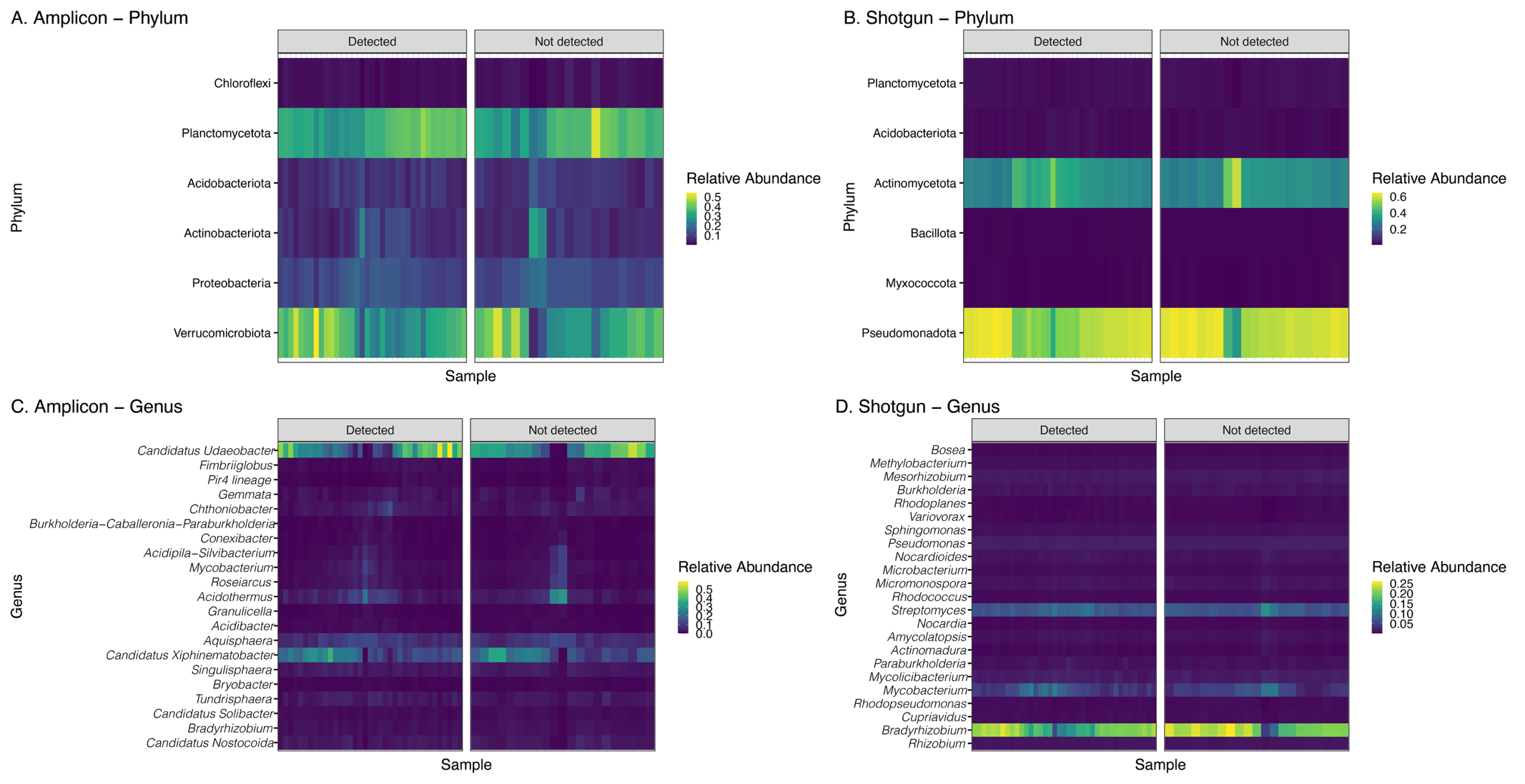


Figure S5: Heatmap of the relative abundance of the top bacterial phyla (>1% MRA), and genera (>0.5% MRA) identified using amplicon and shotgun metagenomic sequencing. Detection of *P. agathidicida* was determined by LAMP analysis (detected *n* = 37 (amplicon), 39 (shotgun), or not detected (*n* = 21)). The heatmap uses NMDS ordination to order the samples (columns) arranging them based on their similarity in microbial community composition as captured by the first ordination axis.


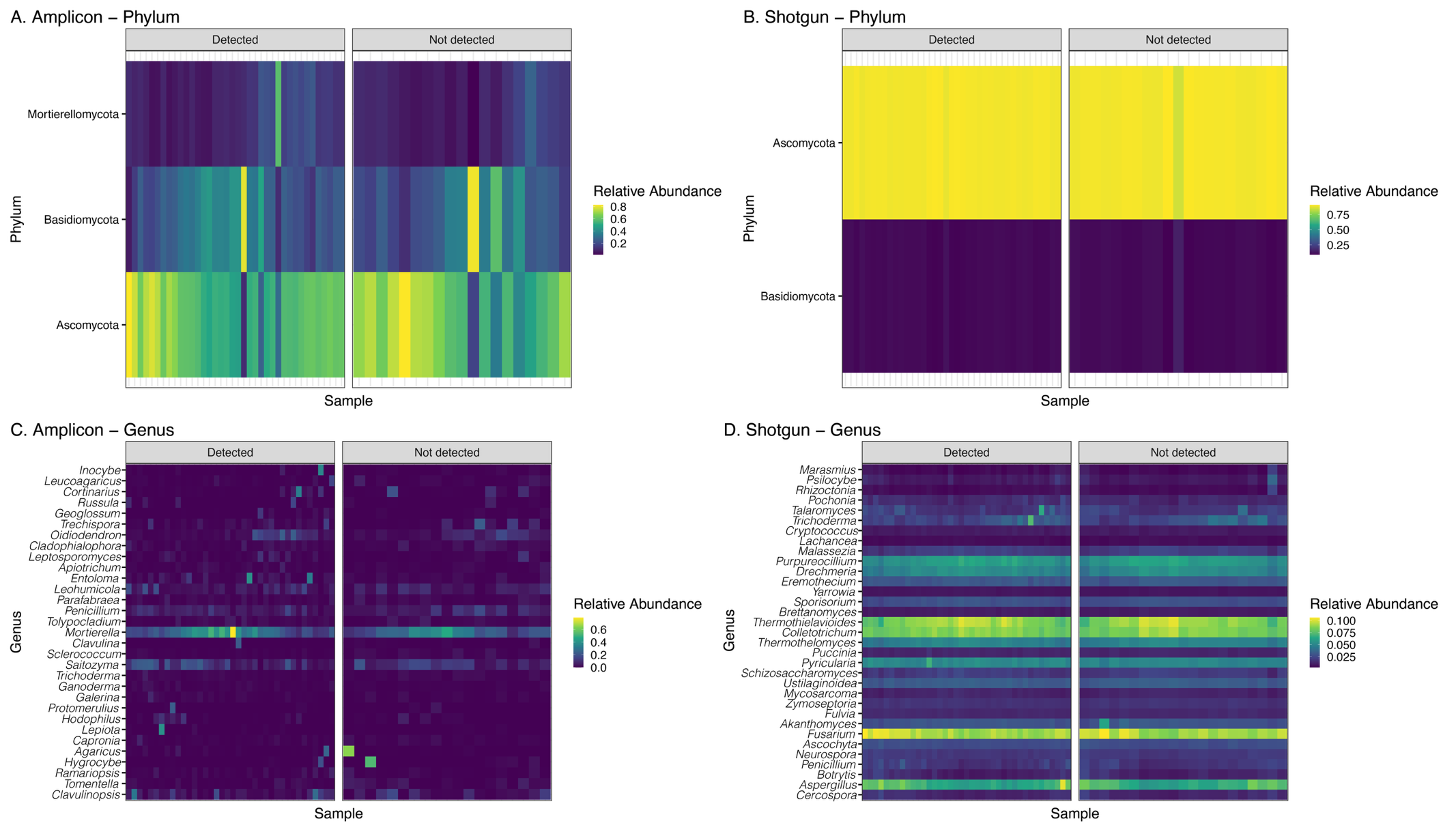


Figure S6: Heatmap of the relative abundance of the top fungal phyla (>1% MRA), and genera (>0.5% MRA) identified using amplicon and shotgun metagenomic sequencing. Detection of *P. agathidicida* was determined by LAMP analysis (detected *n* = 38 (amplicon), 39 (shotgun), or not detected *n* = 19 (amplicon), 21 (shotgun)). The heatmap uses NMDS ordination to order the samples (columns) arranging them based on their similarity in microbial community composition as captured by the first ordination axis.

Figure S7: Principal Coordinates Analysis (PCoA) of bacterial (A and B) and fungal (C and D) genus-level community composition of Bray-Curtis distance matrices from amplicon and shotgun metagenome datasets. Samples were normalised by cumulative-sum scaling. Points are coloured based on LAMP detection of *P. agathidicida* (Bacterial dataset: detected *n* = 37 (amplicon), 39 (shotgun) and not detected *n* = 21 (amplicon and shotgun). Fungal dataset: detected *n* = 38 (amplicon), 39 (shotgun), or not detected *n* = 19 (amplicon), 21 (shotgun).

Figure S8: Measures of bacterial (A and B) and fungal (C and D) taxonomic alpha diversity at the genus level estimated by Shannon diversity and observed richness from amplicon and shotgun metagenome datasets. Samples are grouped based on the LAMP detection of *P. agathidicida* (Bacterial dataset: detected *n* = 37 (amplicon), 39 (shotgun) and not detected *n* = 21 (amplicon and shotgun). Fungal dataset: detected *n* = 38 (amplicon), 39 (shotgun), or not detected *n* = 19 (amplicon), 21 (shotgun)). Samples were rarefied to an even depth (Reads per sample: amplicon 16S: 15,420, amplicon ITS: 4,192, shotgun bacteria: 9,963,524, shotgun fungi: 42,440). Boxes represent the interquartile range of the data (25^th^ and 75^th^ percentiles), whiskers show the largest and smallest values 1.5x the IQR and median values are represented by the bar within each box.


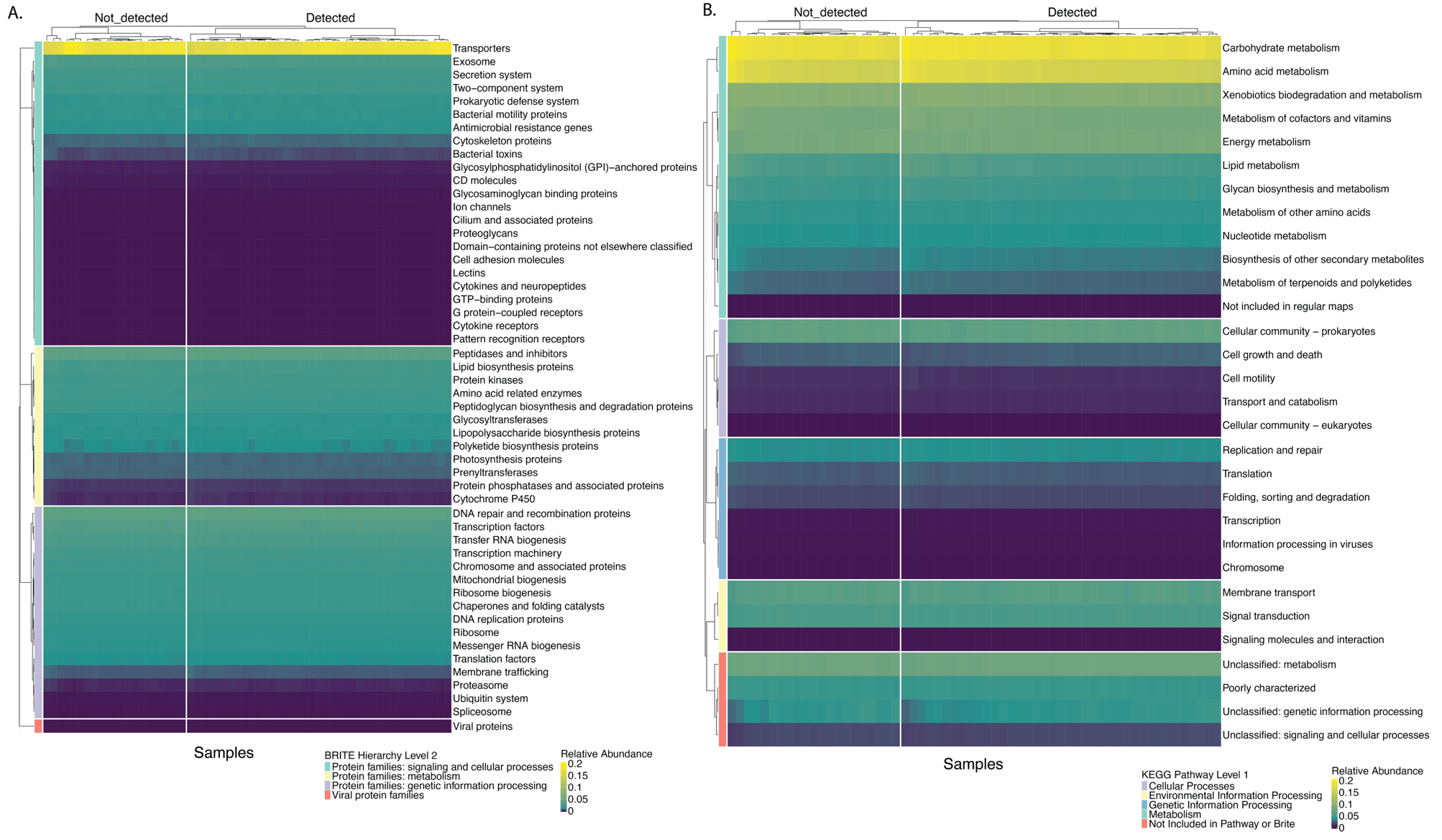


Figure S9: Heatmaps showing the relative abundance of KOs identified through shotgun metagenome sequencing and eggNOG annotation, grouped by KEGG functional classifications and LAMP detection of *P. agathidicida* (detected *n* = 39, not detected *n* = 21). (A) KOs grouped according to KEGG BRITE hierarchies at level 2 and level 3, (B) KOs grouped by KEGG pathway levels 1 and level 2. Samples are clustered based on default Euclidian clustering from the ComplexHeatmap R package. Pathways associated with Organismal systems and Human disease were removed from the dataset.


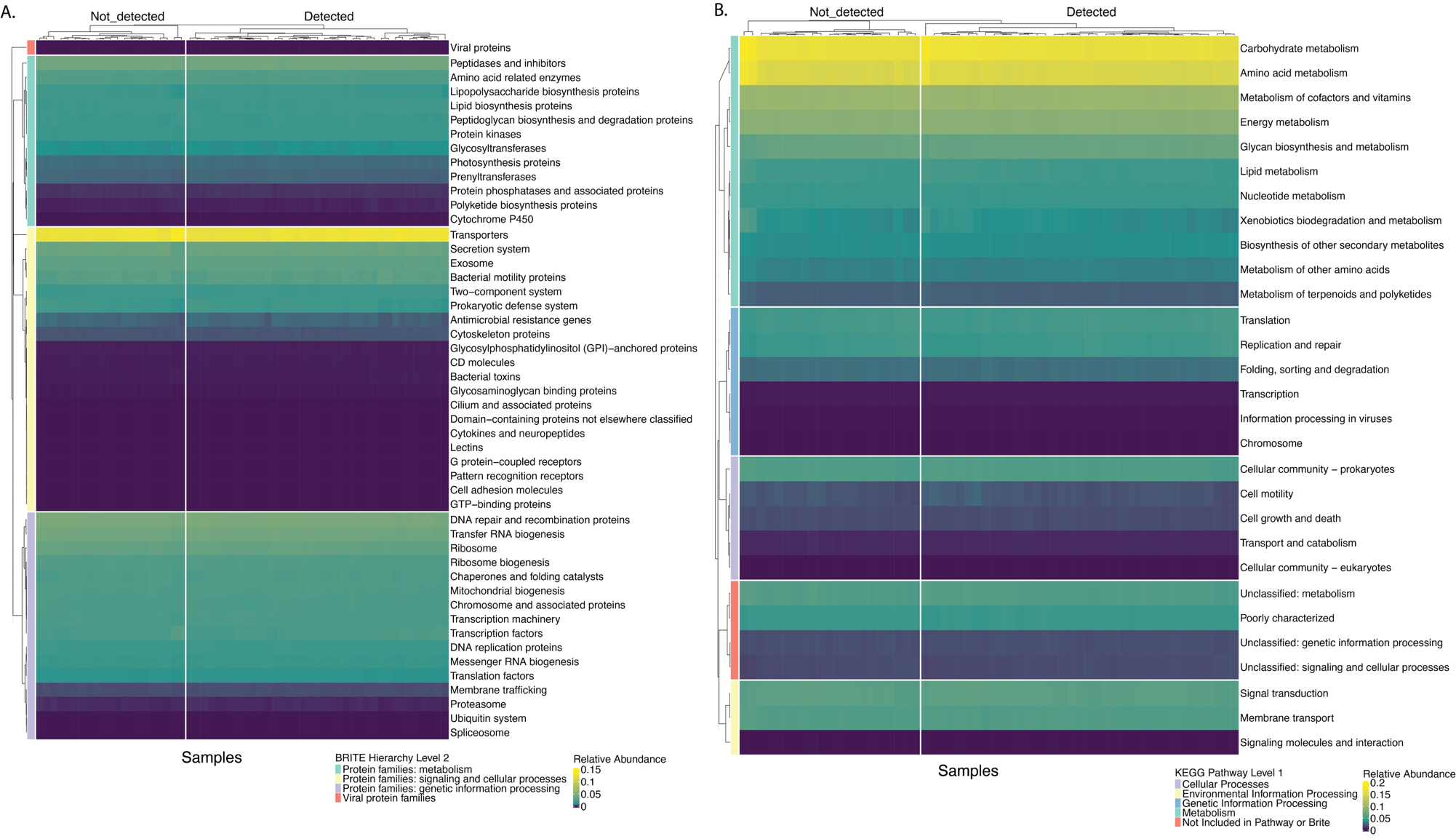


Figure S10: Heatmaps showing the relative abundance of KOs identified through functional inference by PICRUSt2 of ASVs from amplicon sequencing, grouped by KEGG functional classifications and LAMP detection of *P. agathidicida* (detected *n* = 37, not detected *n* = 21). (A) KOs grouped according to KEGG BRITE hierarchies at level 2 and level 3, (B) KOs grouped by KEGG pathway levels 1 and level 2. Samples are clustered based on default Euclidian clustering from the ComplexHeatmap R package. Pathways associated with Organismal systems and Human disease were removed from the dataset.

Figure S11: Boxplots showing estimated alpha diversity of microbial functional potential based on KO profiles from (A) amplicon sequencing (via PICRUSt2) and (B) shotgun metagenome sequencing (via eggNOG annotation). Samples were grouped by LAMP detection of *P. agathidicida* (detected *n* = 37 (amplicon), 39 (shotgun), not detected *n* = 21 (amplicon), 21 (shotgun)). Boxes represent the interquartile range of the data (25^th^ and 75^th^ percentiles), whiskers show the largest and smallest values 1.5x the IQR and median values are represented by the bar within each box.

Figure S12: Principal Coordinates Analysis (PCoA) of amplicon-based inference (PICRUSt2) and shotgun metagenome sequencing (eggNOG annotation) of KOs based on Bray-Curtis distance matrices. Samples were normalised by CSS. Points are coloured based on LAMP detection of *P. agathidicida* (detected *n* = 37 (amplicon), 39 (shotgun), not detected *n* = 21 (amplicon), 21 (shotgun)).

Figure S13: Venn diagram showing the overlap of KOs identified from shotgun metagenome sequencing (eggNOG annotation) and amplicon-based functional inference (PICRUSt2).
